# An R package that automatically collects and archives details for reproducible computing

**DOI:** 10.1186/1471-2105-15-138

**Published:** 2014-05-10

**Authors:** Zhifa Liu, Stan Pounds

**Affiliations:** 1 Department of Biostatistics, St Jude Children’s Research Hospital, Memphis TN 38105, USA

## Abstract

**Background:**

It is scientifically and ethically imperative that the results of statistical analysis of biomedical research data be computationally reproducible in the sense that the reported results can be easily recapitulated from the study data. Some statistical analyses are computationally a function of many data files, program files, and other details that are updated or corrected over time. In many applications, it is infeasible to manually maintain an accurate and complete record of all these details about a particular analysis.

**Results:**

Therefore, we developed the rctrack package that automatically collects and archives *read only* copies of program files, data files, and other details needed to computationally reproduce an analysis.

**Conclusions:**

The rctrack package uses the trace function to temporarily embed detail collection procedures into functions that read files, write files, or generate random numbers so that no special modifications of the primary R program are necessary. At the conclusion of the analysis, rctrack uses these details to automatically generate a *read only* archive of data files, program files, result files, and other details needed to recapitulate the analysis results. Information about this archive may be included as an appendix of a report generated by Sweave or knitR. Here, we describe the usage, implementation, and other features of the rctrack package. The rctrack package is freely available from http://www.stjuderesearch.org/site/depts/biostats/rctrack under the GPL license.

## Background

The ability to reproduce research results is a cornerstone of the scientific method. Research results that are not reproducible are generally considered invalid by the scientific community. The result of a laboratory investigation must be independently reproduced by others to be considered valid. Similarly, the validity of a clinical research finding is established via recapitulation in multiple distinct clinical research cohorts.

The exciting advances in data collection biotechnologies (microarrays, sequencing, proteomics, etc) present the biomedical research community with special challenges related to data interpretation and reproducibility of research results. The interpretation of massive data sets requires the use of sophisticated computational and statistical analysis methods. The data sets themselves evolve as new technologies are introduced, additional tissue samples become available, clinical follow-up is updated, etc. A specific analysis result may be a function of dozens of data files, program files, and other details. Without carefully tracking details about how those files are used to generate a specific result, it can be challenging if not impossible to describe how any particular research result can be recapitulated from the study data. This challenge represents a crisis for reproducibility as a pillar of the scientific method. Consequently, some journals are developing policies to encourage computational reproducibility [1] and federal authority to audit the computational reproducibility of research results is being expanded [2]. Most recently, the National Cancer Institute issued guidelines that strongly encourage investigators to maintain high standards of computational reproducibility in their ‘omics’ studies [3,4].

Also, some recent mishaps in clinical cancer genomics research have shown that there is an ethical obligation to ensure that computational results are fully reproducible. The first indication of problems in a series of clinical trials was the inability to computationally reproduce the results of the supporting scientific publications from the publicly available data sets [5,6]. Investigative follow-up of the analysis result discrepancies showed that the supporting science was fundamentally flawed [7]. Subsequently, the publications have been retracted and the clinical trials closed [8]. Thus, it is ethically imperative that data analysis results be computationally reproducible before they are used to direct therapy in a clinical trial [2,9].

Several computational tools have been developed to facilitate reproducible computing [10]. For example, *Sweave*[11] uses R (http://www.r-project.org) to compute statistical results and inserts them into a LATE X (http://www.latex-project.org/) typesetting program that is subsequently converted into a PDF report. To enable this functionality, Sweave defines a special syntax to switch between R code and LATE X code within the same file. In this way, the Sweave program file directly documents the top-level R code used to generate the corresponding PDF report. Similarly, the R packages knitr [12] and lazyWeave [13] enable one to computationally insert results determined with R into a Wiki mark-up file and an open office document file, respectively. These literate programming [14] systems internally document exactly how the results in the report file were produced. These literate programming environments significantly enhance the scientific community’s ability to perform reproducible computing.

However, literate programming systems fail to address other challenges to reproducible computing. As previously mentioned, one analysis result may be a function of a very large number of input data files (such as microarray image files, sequence alignment files, etc) and computer program files (such as the primary analysis program, some locally developed R routines, specific versions of R packages, etc). These files must be collected and archived to ensure that the result may be reproduced at a later date. Manually reading through a program to identify and collect all those files is very tedious and cumbersome. Thus, there is a need to computationally collect and archive those files.

Some tools have been developed to support reproducible computing by automatically archiving files. CDE software (http://www.pgbovine.net/cde.html) automatically generates an archive folder that replicates the entire directory structure for every file used to execute a specific Linux command so that the identical command can be executed on another Linux machine without any conflicts [15]. The technical completeness of the CDE archive is very impressive and CDE is very helpful for many Linux users who wish to ensure the computational reproducibility of their work.

Nevertheless, CDE has several limitations and drawbacks. Obviously, CDE is not helpful for people who use operating systems other than Linux and Unix. Also, the archive folder of CDE is excessively redundant for some settings. For example, the CDE archive includes a copy of many installation files for every software program (R, MatLab, SAS, Java, etc) used to perform an analysis. As CDE is used to document many analyses for multiple projects by a group of users that all have access to the same programs (such as a department of biostatistics or computational bioloy), the redundancy of such program files in CDE archives will begin to unnecessarily strain the storage resources of the computing infrastructure. This redundancy is amplified when the same large data files (such as genotype data files for genome-wide association studies) are present in multiple CDE archives. Additionally, collecting a large number of copies of proprietary software files in many CDE archives may inadvertently pose some legal problems. Furtermore, although the CDE archive is complete from a technical computing perspective, it still does not necessarily contain all the information necessary to exactly recapitulate the results of statistical analysis procedures such as bootstrapping, permutation, and simulation that rely on random number generation [16]. The initial seed and the random number generator must be retained for such analyses to be fully reproducible and such information will not be stored in the CDE archive if the program files did not explicitly set the value for the initial seed. Finally, CDE does not automatically generate any file to help a reviewer to understand the computational relationships among files such as indicating that specific program files generated specific result files. Understanding such computational relationships is necessary to critically evaluate the appropriateness of the methods and the scientific validity of the results.

We have developed the package **rctrack** (Additional file 1, also available from our website listed below) to automatically archive data files, code files, and other details to support reproducible computing for R software that is widely used for statistical analysis of biomedical data and is freely available for the Unix/Linux, Windows, and MacOS operating systems. The **rctrack** package may be used in conjunction with literate programming R packages (Sweave, knitR) and does not require the user to modify previously written R programs. It automatically documents details regarding random number generation and a sketch of the R function call stack at the time that data, code, or result files are accessed or generated. These details are saved in an archive that includes additional files that have been automatically archived according to default or user-specified parameters to control the contents and size of the archive. The files in this archive can be used to develop a custom R package or other set of files to help others recapitulate the analysis results at a later date. Finally, the rctrack package also provides functions to audit one archive and to compare two archives at a later date.

## Implementation

**Figure 1:**
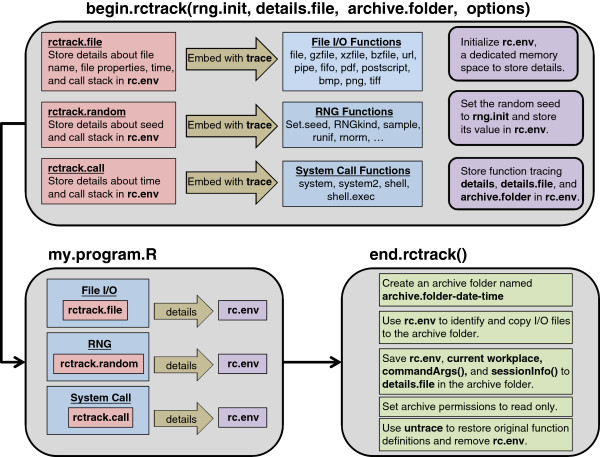
Implementation of rctrack package.

Figure 1 illustrates the implementation of the rctrack package. The function begin.rctrack sets the initial value of the seed for random number generation, creates a dedicated memory environment rc.env to store details that are subsequently collected, and issues a series of trace statements that temporarily *embed* a set of detail-collection procedures into the *definitions* of R functions that access files for reading/writing, generate random numbers, and issue system calls. The detail-collection procedures are then executed *as part of* the functions that access files, generate random numbers, initialize the graphic devices, and issue system calls until the end.rctrack command is executed. Thus, the detail collection is performed automatically without any special modification of the R program.

After the program execution is complete, the end.rctrack statement creates an archive directory with a time-stamp suffix in its name. The end.rctrack function then accesses the information in rc.env to identify and copy files to this archive directory. The md5sum function is used to compute the 32-byte MD5 checksum for each file and these results are recorded in rc.env. The end.rctrack saves the R sessionInfo(), commandArgs(), and the contents of rc.env to an Rdata file in the archive directory. Next, end.rctrack sets the the archive directory permissions to *read only* to prevent the user from inadvertently modifying the archive later. Finally, end.rctrack removes the rc.env environment and uses untrace to restore the original definitions of all the tracked functions.

The rctrack package also includes tools to audit and compare archives at a later time. The rc.check.archive checks the existence, size, MD5 checksum, and modification times of files that are listed in the Rdata file in the archive and reports any discrepancies in those parameters. The rc.compare.archives function compares two archives generated by the rctrack package. The rc.compare.archives function compares the size, MD5 checksums, and modification times of files that are present in each of two archives and also lists files that are present in one archive folder but not the other archive folder. Finally, the rc.check.zipfile function checks the existence, size, MD5 checksum, and modification times of files in an rctrack zip file archive and compares them to the values of those file attributes that were recorded at the time the zip file archive was generated. These auditing functions are documented in the package.

### Main features

Table 1 lists optional arguments of begin.rctrack that govern the detail collection and archiving. We anticipate that the rng.init, details.file, and archive.folder options will be of greatest interest to most users. The rng.init argument dictates the random number generator to be used and the initial seed for random number generation. The default seed is 123456789 and the default generator is R’s default generator. The default of details.file is “rc.details.Rdata”. The details will be saved to this file when end.rctrack is called. The default of archive.folder is NULL. With these defaults, an archive folder with a name of the form YYYY-MM-DD-HH-MM-SS (year-month-day-hour-minute-second) will be created in the present working directory at the time that end.rctrack is called. Otherwise, the name of the archive folder will have the form archive.folder-YYYY-MM-DD-HH-MM-SS. The details.file and copies of other files will be saved in this archive directory when end.rctrack is called. We provide defaults for rng.init, details.file and archive.folder for user convenience but nevertheless recommend explicitly setting the values of these arguments in most applications.

**Table 1:** Optional arguments of begin.rctrack

**Argument**	**Value**	**Action**
rng.init	a list with components	specifies how random number generation
	seed, kind, and normal.kind	will be initialized by set.seed
details.file	name of Rdata file to store details	end.rctrack will save details to this file
archive.folder	character string name of	end.rctrack will create a directory
	the archive directory	with name defined by appending a time
		stamp to archive.folder
maxsize.archive	a numeric with file size in bytes	end.rctrack will NOT copy INPUT
		files larger than this size to
		the archive directory
do.not.archive	a vector of character strings	files with extensions matching these strings
		will NOT be moved to the archive directory
skip.file.calls	a vector of character strings	a list of high-level file access statements
	with function names	for which details will NOT be collected
skip.empty.description	a logical (TRUE/FALSE) that	if TRUE, then no details about file
	indicates whether to skip detail	access events with empty descriptions
	collection for empty file descriptions	(no file name) will be collected
rng.trace	a logical that indicates	if TRUE, begin.rctrack will
	whether to collect details	embed rctrack.random into each of the
	about every random number	functions listed in the rng.functions
	generation event	argument so that a record of every call
		to those functions is retained.
rng.functions	a vector of character strings with	begin.rctrack will embed
	the names of functions that	rctrack.random into each of these
	generate random numbers	functions so that their use
		is documented in rc.env and
		details.file
print.trace	a logical that indicates	Messages will be issued if TRUE
	whether to print messages about	
	detail tracking	

There are other options that control the level and extent of detail collection and archiving. These options may be useful in certain settings. For example, the user can set up the maxsize.archive to control the maximum size of an INPUT file to be copied and frozen in the archive folder. This option allows the user to avoid filling their disk space with multiple copies of very large data files, such as genotype data files for a genome-wide association study. The do.not.archive option also controls the automatic file archiving. This option specifies a character vector of file name extensions. Files with these extensions will not be automatically archived. This may be useful to prevent repeated archiving of individual genotype or expression signal files (such as an Affymetrix CEL file). The skip.file.calls option is a vector of character strings with function names. No details about files accessed through calls to these functions will be collected nor will files accessed through calls to these functions be archived. This option prevents the archive from being cluttered with many low-level files accessed by loading R packages with the library or require statements. Details about the usage of R packages are captured separately through saving the results of the sessionInfo() command. Likewise, the option skip.empty.description prevents the collection of many unnecessary details about some technical low-level calls to the file function that do not actually read or write files. The rng.trace option indicates whether or not to retain a record of every random number generation event. The rng.trace option defaults to true. However, for some applications in some computing environments, users may want to set rng.trace to FALSE so that calculations which are intensive in their use of random number can be more rapidly completed. The rng.functions option accepts a character vector of names of functions that generate random numbers. This is the list of random number generating functions that will be traced (if rng.trace is TRUE) from the begin.rctrack statement until the end.rctrack statement. Finally, the print.trace option controls whether messages regarding the collection of reproducible computing details are issued while the program is running. Defaults have been provided for all of these options.

Sometimes, R may be used to issue system commands to perform some calculation with other software (MatLab, PLINK, etc). The rctrack package will note that such an action has occurred but is currently unable to collect details about the actions of those other softwares. In some cases, it may be possible to write a wrapper function in R that will add information about what the other software does to the collection of details collected by rctrack (such as the input files and output files of the external software program). In other cases, it can be useful to have a record indicating that these actions have occurred so that the user can manually identify and archive the necessary details.

### Usage

**Figure 2:**
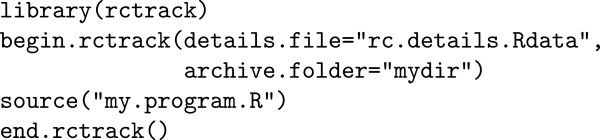
Use of rctrack package.

The rctrack package is extremely simple to use; it only requires loading the library and issuing begin.rctrack to start collecting details and issuing end.rctrack to save the details to an Rdata file, create a read only archive of the files used in the data analysis, and discontinue detail collection. Figure 2 shows how to use the rctrack package to track the computing details of an R program.

In this example, R will collect details about every instance that my.program.R and all of its subprograms read or write a file, generate random numbers, or issue a system call. All these details, the starting value of the seed for random number generation, and the session information will be saved in a read-only file mydir-date-time/rc.details.Rdata, where date-time is a date-time stamp appended to the name of the archive directory mydir. Additionally, read-only copies of all input files below a specified size and all output files (regardless of size) will be placed in the archive directory mydir-date-time. (The permissions are automatically set to read-only to prevent the user from inadvertently deleting or modifying the archived files; it does not prevent the deliberate modification of archived files because the read-only permissions can be modified later.) Thus, mydir-date-time provides a complete record of the calculations performed by my.program.R at the date and time indicated in the directory name. The date-time stamp serves to identify specific versions of the analysis. Most importantly, **no** modifications need to be made to my.program.R for these detail tracking and archiving operations to occur. Thus, rctrack provides a comprehensive solution for collecting and archiving reproducible computing details while minimizing user burden.

## Results

**Figure 3:**
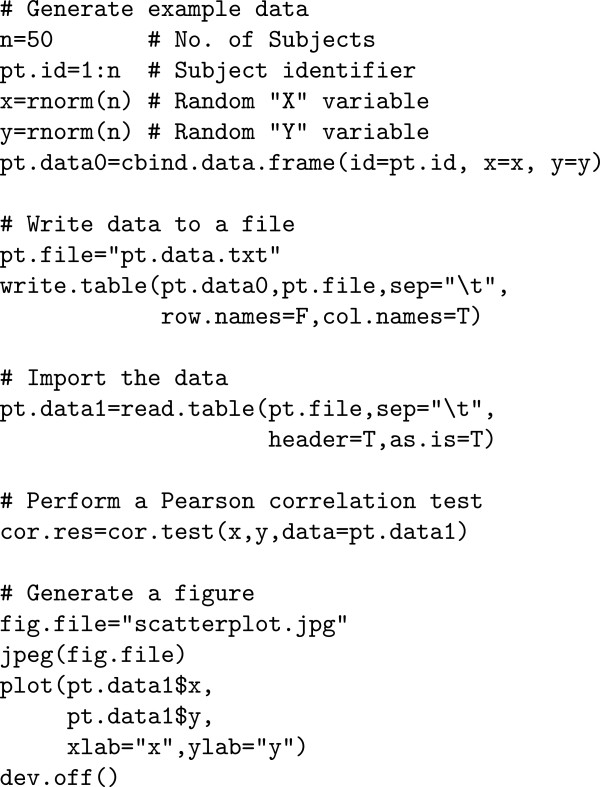
**Contents of *my.program.R * from Figure **2**.**

Figure 3 provides an illustrative example of the contents of *my.program.R* of Figure 2. The example includes random number generation (rnorm), writing a tabular output file (write.table), reading a tabular input file (read.table), and generating a graphical output file (jpeg).

**Figure 4:**
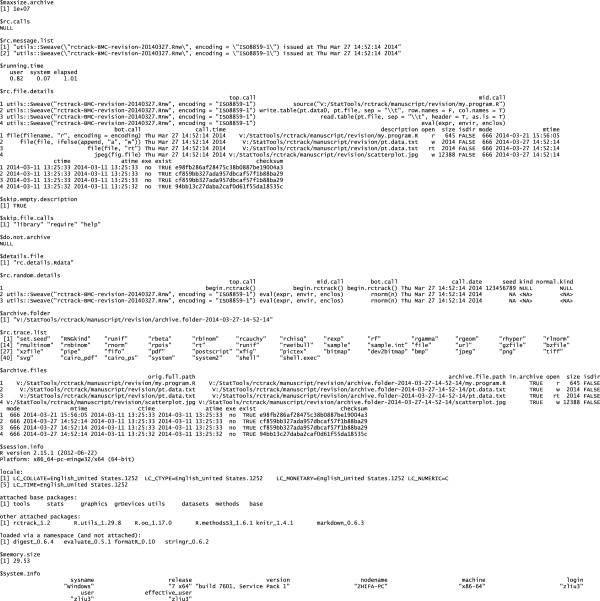
Appendix produced by rctrack.

Table 2 shows an abbreviated version of the contents of the rc.file.details object that was stored in the rc.env environment and then saved to rc.details.Rdata. The rc.file.details object is a data.frame that contains a sketch of the call stack at the time a file was accessed (in the columns top.call, mid.call, and bot.call), the time the traced file input/output command was called, the file description (usually a file name), and how the file was opened (read or write). The full version contains the entire function calls and the full file path in the description (Figure 4). In this example, the file details indicate that the files were accessed during a call to Sweave (to generate this paper), which subsequently called read.table, write.table, or jpeg.

**Table 2:** Abbreviated contents of rc.file.details

	**top.call**	**mid.call**	**bot.call**	**call.time**	**description**	**open**
1	Sweave	source	file	Thu Mar 27 14:52:14 2014	my.program.R	r
2	Sweave	write.table	file	Thu Mar 27 14:52:14 2014	pt.data.txt	w
3	Sweave	read.table	file	Thu Mar 27 14:52:14 2014	pt.data.txt	rt
4	Sweave	eval	jpeg	Thu Mar 27 14:52:14 2014	scatterplot.jpg	w

Also, Table 3 shows an abbreviated version of the contents of the random.details object that was stored in the rc.env environment and then saved to the file rc.details.Rdata. Again, the random.details object is a data.frame that contains a sketch of the call stack at the time a random number generation operation occurred. The random.details objective also contains the starting value of the random seed and indicates what random number generator was used. The top row indicates that the seed was set by begin.rctrack and subsequent rows are records of how rnorm was used to generate the data. The full version contains complete function calls in the sketch of the call stack.

**Table 3:** Abbreviated contents of rc.random.details

	**top.call**	**mid.call**	**bot.call**	**call.date**	** seed**	**kind**	**normal.kind**
1	begin.rctrack	begin.rctrack	begin.rctrack	Mar 27 14:52:14	123456789	NULL	NULL
2	Sweave	eval	rnorm	Mar 27 14:52:14			
3	Sweave	eval	rnorm	Mar 27 14:52:14			

Finally, the file rc.details.Rdata contains additional information that may be useful for documenting and achieving reproducible computing. In addition to all the information collected in the special rc.env memory environment, it contains the R sessionInfo(), Sys.info(), and memory.size() at the time end.rctrack was called. The Figure 4 shows the complete contents of rc.details.Rdata for the example shown by Figures 2 and 3. Additional file 2 is the archive generated by this example; Additional file 3 provides the program files and gives detailed instructions for how to execute this example.

We also ran the simple example *my.program.R* in Figure 3 under CDE. The CDE archive was much larger than the rctrack archive. The rctrack archive contained 8 files in one folder with a total size of 96 KB. The CDE archive contained 249 files in 207 folders with a total size of 173 MB, which is over 1,000 times larger than the rctrack archive. The bulk of the CDE archive contained environment files, shared libraries, R installation files, and R package files that are redundant for R users who already have those files or can easily obtain them. The exhaustive CDE archive does provide the advantage that others who do not have these R libraries can issue a single Linux command to repcapitulate the calculation within random number generation variability. The rctrack provides sufficient information for others who already have R and the necessary libraries to recapitulate the calculation with some modest efforts (such as installing the packages and revising the file paths in the code file). The rctrack package also documents the initial random seed and random number generator so that the calculation can be exactly recapitulated. Furthermore, any Linux users who wish to have all the information captured by both CDE and rctrack may run rctrack under CDE.

## Discussion

Reproducible computing is an essential component of biomedical research in the ‘big data’ era. Literate programming systems such as Sweave and knitr internally document specific calculations at the top-level program code level. However, to completely achieve complete permanent reproducibility, one must identify and archive *all* data files and other computational components of an analysis. The rctrack package provides a simple and effective computational approach to collect and archive the plethora of low-level details needed to achieve and document complete and permanent reproducibility of a statistical analysis. In particular, the analysis archive can be provided as Additional files of a published report to provide complete documentation for researchers, reviewers, supervisors, institutions, or regulatory agencies who are interested in recapitulating the analysis results or using the analysis methods in their own studies. A custom R package is an excellent format in which to distribute these suppelementary materials; the rctrack package can help the user to create such a package by assembling all the necessary elements into a single archive file. This should greatly enhance the rigor and transparency of scientific discourse and expedite the evaluation and adoption of robust data analysis methodologies.

Unlike CDE, the rctrack package captures the intial seed for random number generation. Many statistical analysis procedures such as permutation, bootstrap, and Markov chain Monte Carlo simulation rely explicitly on random number generation. The rctrack package captures sufficient information to recapitulate random number generation with the stats package on a single processor and to recapitulate parallel random number generation as implemented in the doRNG package. The rctrack package does not capture all seeds in the parallel implementation in doRNG but still captures the initial seed which is sufficient information to recapitulate the random number series. The rctrack package only *monitors* random number generation, it does not attempt to *modify* or otherwise *control* random number generation. Thus, rctrack will not introduce any problems for random number generation that is properly implemented in a serial or parallel manner.

The current version of the rctrack package has some limitations that we wish to address in future versions. As previously mentioned, the user must modify the file paths in the archive to recapitulate the analysis. The rctrack package captures the complete original absolute file path and the archived file path for every file. We plan to use this information in a new version of the software that alleviates the user of the burden of manually modifying the file paths. We will accomplish this by using the information to computationally redirect file paths. The current version does not collect details or archive files for calculations that were performed by external software via a system call from R but instead simply notes that system calls to external software were performed. We plan to explore and develop ways to use the Linux ptrace command and analogous features in Windows to capture relevant information from external software calls made via the R system command. Also, the current version of rctrack does not combine information from multiple R sessions that run independently in parallel. We are currently developing routines that will combine information from such an arrray of R sessions.

## Conclusion

The rctrack package was developed to minimize user burden so that it can be immediately useful in practice. The rctrack package only requires statements to initiate and terminate collection of details for reproducible computing; no additional programming effort is required. Furthermore, the rctrack package may be used in conjunction with the literate programming packages Sweave, knitR, and lazyweave so that the reproducible computing details may be incorporated as an appendix of the reports generated by those tools. Finally, the open source rctrack package provides a roadmap for advanced users to expand the capabilities to collect and archive other details about their specific calculations. Therefore, rctrack will be very useful in the modern era of ‘big data’.

## Availability and requirements

**Project name:** rctrack **Project Homepage: **http://www.stjuderesearch.org/site/depts/biostats/rctrack**Operating System:** Platform independent **Other requirement:** R 2.15.0 or higher **License:** GPL

## Competing interests

The authors above declare that they have no competing interests.

## Authors’ contributions

Dr. Pounds and Mr. Liu designed the study, developed and documented the software, and drafted the manuscript. All authors read and approved the final manuscript.

## Supplementary Material

Additional file 1 **rctrack package.** This is the rctrack package. Installation instructions are included in the file *example and instruction.rar*. Click here for file

Additional file 2 **Archive folder for the results.** Archive folder generated by the *rctrack* package for the program *my.program.R* shown in Figure 3. Click here for file

Additional file 3 **Example and instruction.** This file includes the instructions for installing the *rctrack* package and how to perform the example shown in Figure 2. Click here for file
